# Can Cultural Intelligence Affect Employee’s Innovative Behavior? Evidence From Chinese Migrant Workers in South Korea

**DOI:** 10.3389/fpsyg.2020.559246

**Published:** 2020-09-22

**Authors:** Peng Fan, Yixiao Song, Surya Nepal, HyoungTaek Lee

**Affiliations:** ^1^ Department of International Business and Management, School of Economics and Management, Dongguan University of Technology, Dongguan, China; ^2^ Department of Human Resource Management, School of Business Administration, Guangdong University of Finance and Economics, Guangdong, China; ^3^ Department of Business Administration, Changwon National University, Changwon, South Korea; ^4^ Department of International Business, Chungbuk National University, Cheongju, South Korea

**Keywords:** Chinese migrant workers, climate for inclusion, cultural intelligence, knowledge sharing, innovative behavior

## Abstract

This empirical study explores the effect of cultural intelligence (CQ) on migrant workers’ innovative behavior, as well as the mediating role of knowledge sharing on the CQ-innovative behavior relationship. Besides, it also examines the extent to which the mediating process is moderated by climate for inclusion. Using survey data collected from Chinese migrant workers and their supervisors working in South Korea (*n* = 386), migrant workers’ CQ is found to positively impact their innovative behavior through enhanced knowledge sharing. However, it is observed that this indirect relationship is significant, only for migrant workers in a strong climate for inclusion. Thus, these findings reveal the underlying mediation and moderation mechanism and consequently unveil the important role of migrant workers’ CQ in shaping their behavior. This study provides insightful and practical implications to a multicultural organization, where culturally diverse migrant workers work together.

## Introduction

The mobilization of human and nonhuman resources is one of the important developments in the world. A sharp rise in workforce mobility has been observed, especially in Asian countries (Chen, 2015). South Korea, for example, is one of the major destinations for many Asian migrant workers. It is documented that approximately 41.1% (*n* = 215,665) of the migrant workers in South Korea were of Chinese nationality in September 2018 (Lee and Cho, 2019). If properly managed, the cross-cultural diversity that the workforce mobility brings to organizations can provide a variety of information and resources for innovation by employing effective interaction (Ritter and Gemünden, 2004; Fang et al., 2018; Giorgi et al., 2020). However, it is also likely to create barriers to understanding and communication among members and make it more difficult for them to be innovative, due to the knowledge gap between cultures (Tidd and Bessant, 2009; Fang et al., 2018; Giorgi et al., 2020). That is why the identification of the migrants’ competencies, which may affect workers’ interactive behavior, has become a very important subject for both researchers and managers. Cultural intelligence (CQ) refers to “an individual’s capability to deal with culturally diverse situations effectively” (Earley and Ang, 2003, p. 4). Those ones with higher CQ are considered to interact more effectively with individuals outside their own cultures (Jyoti and Kour, 2017). All through the last decade, there has been a proliferation of articles published on CQ (Ott and Michailova, in press). The majority of prior researches that used CQ as an antecedent have connected it with cross-cultural adaption, adjustment, cultural judgment, and expatriate performance (Fang et al., 2018; Rockstuhl and Van Dyne, 2018). In this study, we focus on the concrete ways CQ could enhance migrant workers’ innovative behavior in diverse workplace. This direction is of significance in that the migrant workers, who have to adjust to the new cultural settings, may have unique personal factors and ways to foster their innovative behavior. This study is novel, as it exclusively extends research on the Chinese migrant workers in South Korea. The substantial inflow of Chinese migrant workers into the labor force has rendered it hard for managers in South Korea to ignore the influences of this population on organizational effectiveness and innovation. Because South Korea is an ethnically homogenous society, that its culture emphasizes the blood relationship and the dominant attitudes toward migrant workers has been negative (Choi, 2010). So, it is quite challenging for Chinese migrant workers to cope up with new work environment in South Korea.

This study aims at advancing the understanding regarding the way the migrant workers’ CQ could foster their innovative behavior, considering Chinese migrant workers in South Korea. Prior research has placed emphasis on the fact that CQ could be a catalyst to transform varying cultural perspectives into innovative behavior for the reason that CQ increases the cognitive flexibility (Korzilius et al., 2017). Because CQ assists individuals in overcoming the cultural obstacles, lowering the tension, and mitigating the difficulties faced in the interaction affected by cultural ideology (Jiang et al., 2018), we put forward that these characteristics of CQ make it more comfortable for migrant workers to interact with colleagues and share valuable knowledge in culturally heterogeneous organizations, subsequently facilitating their innovative behavior (Hu and Randel, 2014). Thus, we also consider knowledge sharing as a mediator that plays an important role in the process CQ stimulates migrant workers to adopt innovative behavior. Knowledge sharing is chosen because it has been revealed that high CQ can help eliminate barriers and promote knowledge sharing, which occurs through information exchange and communication between individuals, and then stimulates innovative behavior (Hu and Randel, 2014). In accordance with the trait activation theory (Tett and Burnett, 2003), the functioning of an individual trait is activated by certain social contextual cues. Nonetheless, most previous researches on CQ have been constrained to the beneficial role of CQ in an intercultural setting and overlook the impact of the contextual boundary of organizational environment on CQ and outcomes (Ott and Michailova, in press). For diverse workgroups and organizations, the positive impacts of climate for inclusion have obtained consistent demonstration (Gonzalez and DeNisi, 2009; Dwertmann and Boehm, 2016). Specifically, an organization with a climate of inclusion creates an environment in which “individuals with all different backgrounds – more than just members of historically powerful identity groups – are fairly treated, valued for who they are, and included in core decision-making” (Nishii, 2013, p. 1754). This type of organization is inclined to treat the diversity as an asset and proactively leverages its benefits to develop employees and the organization itself. Consequently, open discussions and knowledge sharing will take place among the members with diverse cultural background, eventually impacting their behaviors and outcomes in this friendly workplace (Ferdman, 2013; Bodla et al., 2018). We take one step ahead for the purpose of investigating whether climate for inclusion is a contextual cue in strengthening the CQ-knowledge sharing-innovation behavior relationship.

This study makes some important contributions. First, the present study places emphasis on the migrant workers’ innovative behavior, thereby responding to the calls for the extension of other outcomes of CQ in the cross-cultural setting (Ott and Michailova, in press). In this manner, we believe it is necessary to improve migrant workers’ CQ, aimed at making them work more effectively in the intercultural workplaces. Second, this study explains the underlying mechanism between migrant workers’ CQ and their innovative behavior and show that the positive effect of CQ on innovative behavior is at least partly due to the increased knowledge sharing (Hu and Randel, 2014; Korzilius et al., 2017). Thus, we suggest that CQ has the potential to make the migrant workers capable of successfully integrating the high-quality informational resources and acquiring support from colleagues by knowledge sharing to foster their innovation. Third, by showcasing that knowledge sharing, the underlying mechanism between migrant workers’ CQ and their innovative behavior may differ by climate for inclusion (Ferdman, 2013; Bodla et al., 2018). We then emphasize the important role of climate for inclusion to enhance the positive indirect effect of CQ on innovative behavior. Furthermore, pivotal practical implications are also carried for human resource (HR) managers in the end of the present study as it documents the conditions under which migrant workers’ high CQ can foster their innovative behavior.

### Relationship Between Cultural Intelligence and Innovative Behavior

Scholars have conducted long-term research on factors that foster intercultural interactions (Gertsen and Søderberg, 2011; Chua et al., 2012). One strategy has been to look for individual characteristics that predict the effective interactions of expatriates, such as personality (Caligiuri, 2000), self-efficacy (Palthe, 2004), and interpersonal skills (Hechanova et al., 2003). Earley and Ang (2003) have integrated most of these views into the concept of CQ. There is evidence that each dimension of CQ can influence intercultural interactions (Chua et al., 2012). As a key capability of adapting to cross cultures, CQ is a reflection of a persons’ adaptability toward intercultural environment (Earley and Ang, 2003). CQ extends the connotation of general intelligence to place emphasis on the people’s capability of communicating with individuals outside their cultures and nations effectively (Jyoti et al., 2019). It was conceptualized as a multidimensional construct comprising metacognitive, cognitive, motivational, and behavioral aspects (Ang et al., 2007; Rockstuhl and Van Dyne, 2018). Despite their qualitative differentiation, these four subcomponents collectively form the overall capability that an individual requires both functioning and managing in intercultural contexts in an effective manner (Earley and Ang, 2003). For such cases, scholars have emphasized the worth of investigating variables at the overall level, putting forward that the mutual and collective functions of the dimensions of a construct are likely to confound or promote the roles of the overall-level construct (Diamantopoulos et al., 2008). That is why, as with the former scholars (e.g., Li et al., 2013; Jiang et al., 2018), we have an interest in the overall-level CQ as well.

A person having high CQ is capable of adapting to the cross-cultures because he/she has the ability to handle novel task and find creative means to solve old problems (Lee and Templer, 2003). Prior research has pointed out that high CQ can promote innovative behavior in multicultural employees as CQ augments the cognitive flexibility (Korzilius et al., 2017). The employee’s innovative behavior, which is stated to be “an employee intentionally introduces or applies new ideas, products, processes, and procedures to his/her work role, work unit, or organization” (Yuan and Woodman, 2010, p. 324), is a valuable asset that could help an organization to stand out in the modern competitive business environment. This kind of behavior can be influenced by the individual factors, as well as organizational factors, such as level of education, individual knowledge sharing, creative self-efficacy, organizational size, structure, organizational knowledge sharing (Kim and Park, 2015; Dy Bunpin et al., 2016), innovation climate (Ren and Zhang, 2015), and HR practices (Prieto and Pérez-Santana, 2014). Actually, employees’ innovative ideas and behavior are not only the products of individuals’ independent thinking but also the outcomes of social interaction among members, so that fluently and openly sharing of diverse and novel ideas and knowledge is key to individual innovation (Perry-Smith and Shalley, 2003). Considering this perspective, interacting with members effectively contributes to boost an individual’s innovative ideas and behavior. Particularly, in a diverse workplace comprising members of different cultures, the capability of interacting effectively becomes extremely important to acquiring information and important resources from each other. On the one hand, employees with higher CQ have a stronger motivation to communicate frequently and effectively with the colleagues from different cultural backgrounds, which could elevate an employee’s central position in the social context, consequently enabling them to acquire diverse information from others (Chen et al., 2008; Afsar et al., in press). Additionally, communicating with colleagues frequently and effectively can also facilitate members to reach consensus on task-related issues, enhance sharing of critical information, and enable the employee’s acquisition of high-quality information (Chen et al., 2008). Acquiring adequate, useful, and latest information spurs employees’ divergent thinking and encourages them to come up with innovative ideas and adopt innovative behavior by integrating various information (Perry-Smith, 2006). Hence, high CQ helps employees succeed in obtaining the informational assistance from colleagues to generate innovative ideas and engage in innovative behavior (Hu et al., 2019). On the other hand, the employees with higher CQ are also capable of understanding other members’ thinking and behavioral pattern better, so that they can actively adjust their mental modes and take appropriate actions to make others feel comfortable and build harmonious interpersonal relationships and an affective support system (Gregory et al., 2009; Ng et al., 2019; Afsar et al., in press). The effective support and encouragement of other members can help improve employees’ confidence and reduce their negative emotions when dealing with novel, risky, and challenging tasks (Liao et al., 2010; Liu et al., 2013). As a result, employees have more likelihood of accomplishing the inventive assignments, putting immense efforts into pursuing the challenging goals, and generating and implementing the innovative thoughts even at bad times (Muñoz-Doyague and Nieto, 2012). A recent longitudinal pilot study also confirmed that CQ training can improve individual innovative work behavior (Azevedo and Shane, 2019). Afsar et al. (in press) also found that CQ could influence individual’s innovative behavior significantly. In this study, we argue that high CQ could bring migrant workers the informational and effective support from colleagues, which is of immense significance and essential for the migrant workers to accomplish challenging tasks, develop novel ideas, and adopt innovative behavior in a culturally diverse workplace (Madjar, 2008). Thus, the following hypothesis is presented:

*Hypothesis* 1: Migrant workers’ CQ is positively related to their innovative behavior.

### The Mediating Role of Knowledge Sharing

Knowledge sharing refers to “the exchange/provision of information and knowledge to help and collaborate with others to solve problems and develop novel ideas” (Cummings, 2004, p. 352), which significantly contributes to the mechanism of generating and implementing of innovative ideas (Grant, 1996; Wang et al., 2017). Innovation is developed when conversations bring together different ideas and knowledge that have never connected previously (Chua et al., 2012). By sharing knowledge, individuals are able to learn and integrate different valuable knowledge, which then facilitates innovative behavior (Srivastava et al., 2006; Mura et al., 2013). Knowledge sharing is associated with the exchange of events, experiences, perceptions, and insights about anything, with the expectation of increasing more understanding as well as insight (Sohail and Daud, 2009), which occurs through information exchange and communication between individuals (Cummings, 2004). Cultural diversity in the organization could influence the process of knowledge sharing. The results of prior studies have indicated that the culture constituted a barrier in the knowledge sharing process because the cross-cultural diversity was likely to give rise to miscommunications and conflicts (De Long and Fahey, 2000; Jyoti et al., 2019). Therefore, it is indeed necessary for culturally diverse members to overcome the cultural barriers for more enriched knowledge sharing. Only those individuals who possess the attributes needed for connecting the various knowledge sources can gain an innovation advantage (Chua et al., 2012). In the cross-cultural interactions, culturally diverse people can expose themselves to knowledge different from their own. The sharing of knowledge and insights with others could lead to a novel combination of knowledge and then foster innovative behavior (Chua et al., 2012). Elenkov and Manev (2009) have asserted that CQ is needed for expatriates to integrate knowledge from colleagues with cultural diversity into their innovative behavior. Lacking CQ may lead to knowledge hiding and conflicts (Bogilović et al., 2017), making innovation more challenging (Afsar et al., in press). Therefore, migrant workers need high CQ to share knowledge with their colleagues in a better manner and integrate knowledge from two or more cultures effectively because they can develop more precise understanding of the cultural scheme and difference (Chen and Lin, 2013; Korzilius et al., 2017). Subsequently, effective knowledge sharing further promotes and implements novel and innovative thoughts (Wang et al., 2017).

Knowledge is a crucial asset for organizations (Nonaka, 1994). Previous research highlights that knowledge sharing among members with different cultural backgrounds is of critical significance to the multicultural organizations’ success (Ang and Inkpen, 2008). Studies have also illustrated the important role of knowledge sharing in supporting and promoting innovation (e.g., Kim and Lee, 2013; Wang et al., 2017). Multicultural work environment may enhance the employees’ innovative knowledge sharing and ultimately stimulate more innovative behavior, when the members are given enough time to work through miscommunications and conflicts (Mishra and Gupta, 2010). In diverse workplaces, the culturally intelligent employees are likely to enjoy successful intercultural interactions with colleagues from different cultural backgrounds (Chen and Lin, 2013). Effective interactions then help promote consensus on task issues, enhance the sharing of pivotal information, and enable the employee’s successful acquisition of high-quality informational resources and support from colleagues to generate novel ideas and engage in innovative behavior (Hu et al., 2019). Moreover, the employees having higher CQ are also capable of better understanding the thinking and behavioral pattern of their colleagues (Gregory et al., 2009), thereby helping build a support system that promotes knowledge sharing to generate new ideas and inspire innovative behavior. Accordingly, we argue that the culturally intelligent migrant worker can more effectively share knowledge with colleagues from different cultures, and further facilitate their innovative activities (Hu and Randel, 2014; Wang et al., 2017). Thus, the following hypothesis is presented:

*Hypothesis* 2: Migrant workers’ CQ indirectly motivates their innovative behavior through the mediation of knowledge sharing.

### The Moderating Role of Climate for Inclusion

In comparison with “diversity climate,” which tends to emphasize the fairness of the personnel practices, as well as the treatment of minority workers, climate for inclusion places broader emphasis on the engagement of whole selves and learning from divergent perspectives (Nishii, 2013). Even though some scholars have examined diversity climate as an aggregated construct, numerous researches still have operationalized individual-level diversity climate, investigating how individual employees’ perceptions of the organizational climate could affect their attitudes and behaviors in the workplace (e.g., McKay and Avery, 2015; Newman et al., 2018). Davies et al. (2019) also used perceived organizational inclusion climate as a resource protecting or detracting factor and explored how it moderated the relationship between resilience and work adjustment in a diverse workplace in their study. Moreover, this study focuses on individual knowledge sharing and innovative behavior rather than import organizational outcomes, so we continue to study climate for inclusion at the individual level. Thus, climate for inclusion is measured on the level to which individual employees perceive that their organizations and managers strive to create an environment, in which employees with diverse backgrounds are fairly treated, valued for who they are, and included in core decision-making processes.

In diverse workgroups and companies, the positive impacts of climate for inclusion have been consistently observed (Gonzalez and DeNisi, 2009). In light of prior climate for inclusion research and trait activation theory (Tett and Burnett, 2003), climate for inclusion is considered as a work contextual cue that can activate the functioning of migrants’ CQ. In a multiculturally friendly setting, where all the employees with diverse cultural background are fairly treated and leverage the full spectrum of their talents (Mor Barak et al., 2016), the inclusive climate offers employees opportunities for cross-cultural interactions, which activates their CQ. Higher CQ then makes them be more proactive in approaching the colleagues with cultural diversity and carefully cater to the requirements of cross-cultural interactions. Moreover, when climate for inclusion exists, social norms do not preclude interactions between migrants and local employees, and then those with higher CQ are more likely to reach out and build effective relationships with others. Effective cross-cultural interactions and relationships bring valuable knowledge to individuals and help them to be innovative. As a result, when employees perceive their organization and managers vigorously advocate an inclusive climate, their own individual CQ will be likely to contribute to knowledge sharing, which then eventually help develop novel ideas and stimulate innovative behavior (Chen and Lin, 2013; Mura et al., 2013; Wang et al., 2017). Conversely, in an organization where a climate for inclusion does not exist, employees hold the belief that their organizations do not treat them fairly, do not value them for who they are, and do not include them in core decision-making. These kinds of perceptions and beliefs may translate into the employees’ negative attitudes toward their colleagues. Furthermore, the boundaries existing among the culturally diverse members cannot be eliminated or even become more obvious. These boundaries separate organization members from each other, limit cross-cultural interactions, and exacerbate mistrust and miscommunication (Bernstein et al., 2010), ultimately leading to an increase in conflict, disengagement, and turnover (Mor Barak et al., 2016). If migrants are generally excluded from intercultural interactions, CQ will not have any impact on knowledge sharing and subsequent innovative behavior. Thus, the positive CQ-knowledge sharing-innovative behavior relationship will be weakened. Considering the above argument together, employees who have higher CQ are more willing to share knowledge at work. By sharing diverse valuable knowledge with colleagues, employees can develop innovative ideas and engage in innovative behavior (Chen and Lin, 2013; Wang et al., 2017). Especially when those culturally intelligent employees have a perception that they are in a strong inclusive climate and feel included in the organization, they will be more willing to communicate frequently with the cultural diverse colleagues and proactively participate in knowledge sharing, which will promote innovations. Hence, we argue that climate for inclusion could enhance the positive impact of migrant workers’ CQ on their innovative behavior *via* knowledge sharing in this study. Thus, the following hypothesis is presented:

*Hypothesis* 3: A climate for inclusion positively moderates the indirect relationship that migrant workers’ CQ has with their innovative behavior *via* knowledge sharing in that the indirect relationship is stronger when the climate for inclusion is stronger.

**Figure 1:**
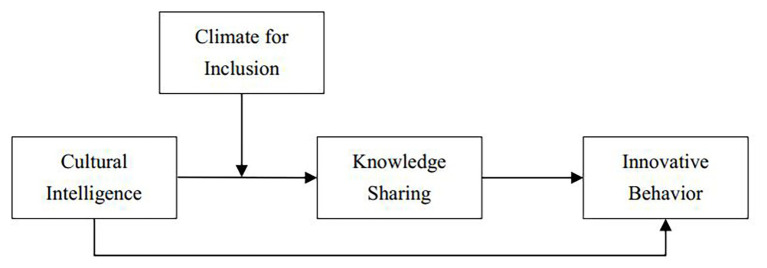
Conceptual model.

Based on the above theoretical analysis, we propose a moderated mediation model to reveal how CQ facilitates migrant workers’ innovative behavior in culturally diverse workforces and the boundary conditions for this kind of effect from climate for inclusion perspective. Climate for inclusion will regulate the first half of knowledge sharing’s path, and the mediating effects on the relationship between CQ and innovative behavior will also be regulated by climate for inclusion. The conceptual model of this study is shown in Figure 1.

## Research Methods

### Sample and Procedure

Following the suggestions by Kim et al. (2016), this study used a snowball sampling technique to recruit the subjects, which has been utilized to target hard-to-reach participants (e.g., migrants). First, the researchers recruited Chinese migrant workers through personal networks of graduate students, alumni, and acquaintances. After showing the purpose of this study, migrant workers who agreed to participate in this research were requested to assist us in collecting the survey. Considering the purpose of this study, we selected participants who had experienced culturally diverse interactions or worked in cross-cultural team. The target population of this study comprised employees and their supervisors from several different industries (trade, cosmetics, retail, and service companies) in South Korea. To ensure the matching of subordinate-supervisor data and evaluate employee’s innovative performance accurately, researchers also encode subordinates and their supervisors during the process. After accomplishing the survey, all participants were given small gifts for their kind help. We designed a two-wave study to control common method bias issue (Podsakoff et al., 2012). At Time 1, a total of 500 employee-rated questionnaires were requested to offer information on demographics and access CQ and climate for inclusion. Four hundred and sixty surveys from employees were returned, with a response rate of 92.0%. At Time 2, employees who had a valid response in previous stage were requested to complete the questionnaire on knowledge sharing *via* paper questionnaires, and the direct supervisors were responsible for evaluating employees’ innovative behavior. In total, 405 responses were returned from employees (81.0%) and their direct supervisors (85.0%). After eliminating nonresponse questionnaires, 386 employee-supervisors dyads responses were received. Employees were nested with 58 direct supervisors (72.5%).

Among the employees, 64.5% were from sale department, 19.4% were from customer service department, and 16.1% were from marketing department; 212 were female (54.7%) and 174 were male (45.3%); 346 (89.6%) were in their 20s and 40 (10.4%) were in 30s; 40.9% have received a bachelor degree and 59.1% held a master’s degree; and their average organizational tenure was 3.35 (*SD* = 1.20) years. For the frequency of cross-cultural interactions, 47.9% of the participants constantly engage in cross-cultural interactions in the workplace, 36.8% of the participants frequently engage in cross-cultural interactions, and 15.8% of the participants occasionally interacted with people with different cultural backgrounds.

### Measures

The scales were initially written in English. As the composition of supervisors included both Chinese and Korean, we distributed supervisor-rated questionnaires with Korean version and employee-rated questionnaires with Chinese version. Because of the nature of this research, eligible Chinese supervisors were those who understood written Korean. To ensure the validation of questionnaires, we applied back-translation method outlined by Brislin (1970). We hired two English-Chinese bilinguals and two English-Korean bilinguals from the university. After performing the translations of employee and supervisor questionnaire surveys following back-translation procedures, researchers then compared the original items with the newly translated version to ensure they had consistent meanings. All items were rated on a 7-point Likert-type scale (1 = strongly disagree and 7 = strongly agree).

### Cultural Intelligence

We used a 20-item scale to access migrant workers’ CQ (Ang et al., 2007). This scale includes four items of metacognitive CQ, six items of cognitive CQ, five items of motivational CQ, and five items of metacognitive CQ. The composite reliabilities of subdimension were 0.87, 0.90, 0.88, and 0.89. As this study focuses on overall CQ, we followed the past study (e.g., Jiang et al., 2018) by conducting a higher-order confirmatory factor analysis (CFA) model to access the validity of CQ as an overall construct. The results presented an acceptable index [*χ* ^2^ = 203.84; *df* = 166; *χ* ^2^/*df* = 1.47, Tucker-Lewis index (TLI) = 0.99, comparative fit index (CFI) = 0.99, root-mean-square error of approximation (RMSEA) = 0.02], indicating the appropriateness of examining CQ at the overall level. Cronbach *α* for the overall CQ was 0.90.

### Knowledge Sharing

Knowledge sharing was accessed by using eight items adapted from Lu et al. (2006). This scale is frequently used by Chinese researchers and mainly assesses within-group knowledge sharing behavior (Zhang et al., 2011). A sample item is “I share with others useful work experience and know-how.” Cronbach *α* for this construct was 0.85.

### Climate for Inclusion

We used 15 items from Nishii (2013) to access climate for inclusion. A sample item is “The performance review process is fair.” The Cronbach *α* for the climate for inclusion scale was 0.88.

### Innovative Behavior

Innovative behavior was evaluated by their supervisors, using six items based on the work of Yuan and Woodman (2010). A sample item is “he/she is innovative.” The Cronbach *α* for this scale was 0.90.

### Control Variables

According to the previous studies (Yuan and Woodman, 2010; Chen and Hou, 2016), we measured education level, gender, and organization tenure to control for the knowledge an employee can draw on to innovate and the employee’s access to organizational resources for conducting innovative behavior.

### Analytical Strategy

Because the data were collected from 32 organizations, this may cause date-nested issue. Before testing our main hypotheses, we have checked for data hierarchy. Following to recommendations by Bliese (2000), we checked the intraclass coefficient correlations if we could use organization-level aggregate scores of climate for inclusion. Reliability of score within group ICC (1) for this scale was 0.05, whereas the reliability of mean group score ICC (2) was 0.42, and less than the benchmark for aggregation (Klein and Kozlowski, 2000). These statistics did not support the aggregation of scores and appropriately reflected the concept of individual-level climate for inclusion.

We tested our hypotheses using SPSS 22.0 software and its macro program PROCESS 2.12 (Hayes, 2013). First, the hierarchical regression analysis was used to examine the direct relationship between CQ and innovative behavior. Second, we applied Model 4 and Model 7 of macro program PROCESS in SPSS to examine the mediation effect and moderated mediation effect. In addition, the bootstrap test was conducted, and the resultant 95% confidence intervals were inspected to examine the significance of mediating effect and moderated mediation effect (Erceg-Hurn and Mirosevich, 2008).

## Results

### Confirmatory Factor Analyses

The validity of the constructs was conducted using CFA with Mplus 7.4. Four variables were employed: CQ, knowledge sharing, climate for inclusion, and innovative behavior. The measurement model revealed the most acceptable fit index (*χ* ^2^/*df* = 1.47, TLI = 0.94, CFI = 0.95, RMSEA = 0.035). The hypothesized measurement model also had a significant difference in *χ* ^2^ over other models (Anderson and Gerbing, 1988; Table 1).

**Table 1:** Results of confirmatory factor analysis for the study variables.

Models	Factors	*χ* ^2^	*df*	▵*χ* ^2^	RMSEA	CFI	TLI
Model 1	CQ, CI, KS, IB	1,637.04	1,114		0.04	0.95	0.94
Model 2	(CQ + CI), KS, IB	6,449.98	1,124	4,812.94^***^	0.11	0.46	0.44
Model 3	(CQ + CI + KS), IB	7,296.95	1,126	5,659.91^***^	0.12	0.38	0.35
Model 4	(CQ + CI + KS + IB)	8,178.16	1,176	6,541.12^***^	0.13	0.29	0.23

CQ, cultural intelligence; KS, knowledge sharing; CI, climate for inclusion; IB, innovative behavior.

*N* = 386.

* *p* < 0.05;

** *p* < 0.01;

*** *p* < 0.001.

### Descriptive Statistics

Results showed that CQ was positively related to knowledge sharing (*r* = 0.27, *p* < 0.01), climate for inclusion (*r* = 0.19, *p* < 0.01), and innovative behavior (*r* = 0.34, *p* < 0.01). Each subdimension of CQ was also positively related to knowledge sharing and innovative behavior. Knowledge sharing was positively related innovative behavior (*r* = 0.46, *p* < 0.01). Climate for inclusion was positively related to innovative behavior (*r* = 0.21, *p* < 0.01; Table 2).

**Table 2:** Means, standard deviations, and correlations among study variables.

	Mean	SD	1	2	3	4	5	6	7	8	9	10
1. Age	27.34	2.00										
2. Tenure	3.35	1.20	0.72^**^									
3. Edu	1.59	0.49	0.09	0.06								
4.MeCQ	4.52	1.34	0.00	0.00	0.01							
5.CoCQ	4.51	1.26	0.04	0.02	0.05	0.38^**^						
6. MoCQ	4.44	1.27	0.02	0.04	0.07	0.40^**^	0.33^**^					
7. BeCQ	4.49	1.27	0.03	0.01	0.01	0.28^**^	0.36^**^	0.35^**^				
8. CQ	4.49	0.92	0.02	0.02	0.04	0.73^**^	0.72^**^	0.73^**^	0.69^**^			
9. KS	4.64	1.09	0.06	0.09	0.04	0.20^**^	0.21^**^	0.15^**^	0.22^**^	0.27^**^		
10. CI	4.52	0.92	0.01	0.06	0.05	0.13^**^	0.11^**^	0.12^*^	0.18^**^	0.19^**^	0.17^**^	
11. IB	4.49	1.28	0.04	0.03	0.07	0.20^**^	0.23^**^	0.26^**^	0.28^**^	0.34^**^	0.46^**^	0.21^**^

*N* = 386. MeCQ, metacognitive CQ; CoCQ, cognitive CQ; MoCQ, motivational CQ; BeCQ, behavioral CQ; CQ, cultural intelligence; KS, knowledge sharing; CI, climate for inclusion; IB, innovative behavior.

* *p* < 0.05;

** *p* < 0.01;

*** *p* < 0.001.

### Hypotheses Testing

Hypothesis 1 proposed that CQ would be positively related to employees’ innovative behavior. As shown in Model 2, we found that CQ was significantly and positively related to innovative behavior (*β* = 0.34, *p* < 0.001). Thus, Hypothesis 1 was supported. Hypothesis 2 predicted that knowledge sharing would mediate the relationship between CQ and innovative behavior. To test Hypothesis 2, we followed the causal steps which developed by Baron and Kenny (1986). When knowledge sharing was included into the regression equation with CQ, the relationship between CQ and innovative behavior decreased slightly (*β* = 0.23, *p* < 0.001), while knowledge sharing was positively related to innovative behavior (*β* = 0.40, *p* < 0.001). The results showed that knowledge sharing partly mediated the CQ-innovative behavior relationship. Additionally, the PROCESS Model 4 for the mediation effect was used to examine the results. The results showed that the indirect effect between CQ and innovative behavior was significant [*β* = 0.15, 95% confidence interval (CI) (0.09, 0.22), excluding zero]. Hypothesis 2 was supported (Table 3).

**Table 3:** Summary of regression results.

	Innovative behavior			Knowledge sharing			
	Model 1	Model 2	Model 3	Model 4	Model 5	Model 6	Model 7
Gender	0.01	0.03	0.03	−0.01	0.01	0.01	0
Age	0.02	0.05	0.05	−0.01	0.02	0.02	0.02
Tenure	0.02	−0.01	−0.04	0.1	0.07	0.06	0.05
Education	0.07	0.05	0.04	0.04	0.03	0.03	0.05
CQ		0.34^***^	0.23^***^		0.27^***^	0.24^***^	0.24^***^
Knowledge sharing			0.40^***^				
Climate for inclusion						0.12	0.14^**^
CQ × CI							0.17^***^
*R* ^2^	0.01	0.12^***^	0.27^***^	0.01	0.08^***^	0.10^***^	0.12^***^
*F*	0.61	10.33^***^	23.13^***^	0.91	6.56^***^	6.56^***^	7.59^***^
Δ*R* ^2^		0.11	0.15		0.07	0.02	0.02

CQ, cultural intelligence; KS, knowledge sharing; CI, climate for inclusion; IB, innovative behavior.

*N* = 386.

* *p* < 0.05;

** *p* < 0.01;

*** *p* < 0.001.

**Figure 2:**
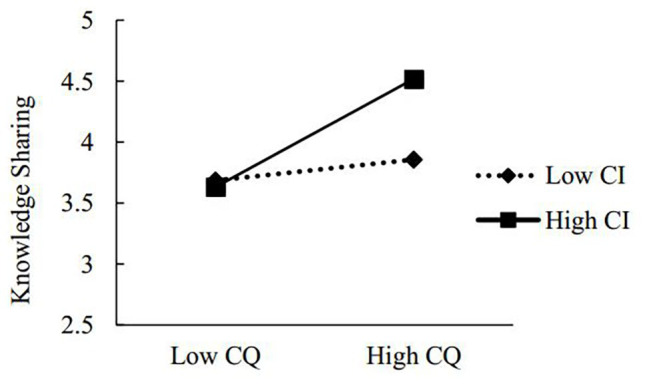
Interactive effects of cultural intelligence (CQ) and climate for inclusion on knowledge sharing.

Hypothesis 3 predicted the mediating relationship between CQ and innovative behavior through knowledge sharing would be moderated by climate for inclusion, and this relationship gets stronger when climate for inclusion is stronger. First, we applied the method outlined by Edwards and Lambert (2007) to test the moderating effect. And we found that climate for inclusion moderates the relationship between CQ and knowledge sharing (*β* = 0.17, *p* < 0.001; Figure 2).

Then, Hayes’ macro program PROCESS in SPSS was adopted to test the moderated mediation effect. As shown in Table 4; at 1 SD above the mean of climate for inclusion, the conditional indirect effect was significant [*β* = 0.23, 95% CI (0.14, 0.32), excluding zero]. In contrast, at 1 SD below the mean, the conditional indirect effect was rather lower and not significant [*β* = 0.05, 95% CI (−0.02, 0.11), including zero]. Overall, these results supported Hypothesis 3.

**Table 4:** Summary of conditional indirect effect.

Moderator = CI	Conditional indirect effect	SE	Boot LLCI	Boot ULCI
Low (−1 SD)	0.05	0.03	−0.02	0.11
High (+1 SD)	0.23	0.04	0.14	0.32
	Bootstrapping effect	SE	Boot LLCI	Boot ULCI
Moderated mediation effect	0.1	0.03	0.05	0.16

*N* = 386. Bootstrap sample size = 5,000.

## Discussion

Although previous studies have strongly suggested the significant role of CQ in culturally dynamic business environments (Jiang et al., 2018), the understanding of the underlying mechanisms of CQ and innovative behavior is unknown. Based on CQ and innovative behavior literature, the research provides an integrated framework on illustrating when and how CQ influenced Chinese migrant worker’s innovative behavior. The results showed that CQ had a positive effect on migrant workers’ innovative behavior, while knowledge sharing mediated the relationship between CQ and innovative behavior. Moreover, climate for inclusion moderated the indirect relationship between CQ and innovative behavior *via* knowledge sharing. In particular, such mediating mechanism was stronger when climate for inclusion was high.

### Theoretical Implication

Our findings provide theoretical contributions to previous researches as well. First, this study contributes to the growing body of research on CQ (Sharma and Hussain, 2017; Ott and Michailova, in press). Numerous studies have investigated the impact of CQ on employee’s behavior, such as job performance (Chen et al., 2010; Jyoti and Kour, 2015), intercultural negotiation (Groves et al., 2015), and decision making (Ang et al., 2007), whereas there is little understanding of how CQ affects nonroutine performance (i.e., innovative behavior) in multicultural contexts. Our findings suggest that an individual with a high level of CQ may retrieve and search relevant information that in turn generates potential ideas with wide possibilities of innovation. In this respect, the current study contributes in addressing this gap and responding to calls for extending other outcomes of CQ in the cross-cultural context (Ott and Michailova, in press). According to the prior study, people who involve a high level of CQ can more easily navigate and understand different cultures, suggesting the generation of novel and useful ideas (Yunlu et al., 2017). The findings of this study are in line with Korzilius et al. (2017), indicating that CQ may help individuals improve their cross-cultural ability and foster innovative outcomes. Hence, the present study enriches the understanding of CQ, a critical cross-cultural ability in culturally dynamic environments.

Second, exploring the mediating effect of knowledge sharing not only provides a conceptual mechanism to explain why culturally intelligent individuals are likely to perform innovative behavior but also uncover the black box of the transmitting process from CQ to innovative behavior. By confirming the mediating role of knowledge sharing in the relationship between CQ and innovative behavior, our study elaborates the prior study (Chua et al., 2012) that CQ is likely to promote employee’s intercultural collaboration, and thus mutually exchange their idea and information. Besides, the result of our study reveals that idea and information sharing can help employees generate innovative behavior (Kim and Park, 2015). Thus, our research enriches knowledge sharing by exploring its antecedent and outcome in multicultural settings. In addition, researchers have examined certain psychological states which could influence innovative behavior, such as intrinsic motivation (Saether, 2019), negative affect (Montani et al., 2018), and psychological empowerment (Afsar and Badir, 2016). Our study extends these studies and identifies generating new ideas and exchanging information based on skills and knowledge should encourage innovative behaviors for migrant workers. The current study represents an effort in responding to the call for facing challenges of knowledge sharing in a globally competitive environment (Asrar-ul-Haq and Anwar, 2016).

Third, the most important implication is the new light shed on the link between climate for inclusion, CQ, knowledge sharing, and innovative behavior. The results show that climate for inclusion could positively strengthen the meditating effect of knowledge sharing in the relationship between CQ and innovative behavior. Thus, our study contributes to extending the inclusion literation by examining the moderating effects of climate for inclusion (Nishii, 2013). In addition, we found that CQ could facilitate migrant workers’ innovative behavior *via* knowledge sharing, only when climate for inclusion was high. Whether CQ translates into innovative behavior through knowledge sharing depends on the boundary condition. High climate for inclusion in organization can offer opportunities for cross-cultural interactions, thus making individual’s CQ relevant for knowledge sharing. In this context, CQ positively affects knowledge sharing, which in turn improves innovative behavior. Conversely, when working in a noninclusive climate, the organizational situation may constrain any intercultural interactions between migrant and local workers. Consequently, CQ will not have any impacts on knowledge sharing and subsequent innovative behaviors. The findings are in line with trait activation theory (Tett and Burnett, 2003), which suggests that intercultural adaptability (i.e., CQ) is more likely to translate into innovative behavior in the inclusive environment when providing situational cues that the knowledge sharing is appropriate. The combination of CQ and climate for inclusion creates a new avenue, motivating employees to share knowledge and improve their innovative behavior. Therefore, the current study provides an integrated framework regarding climate for inclusion as a boundary condition that can establish a favorable environment for enhancing the effectiveness of CQ (Ott and Michailova, in press). As a result, this study highlights the significance of climate for inclusion and provides important insights into why and when CQ matters as predictors of innovative behavior.

### Managerial Implication

In promoting workplace innovation, employees’ innovative behavior has been considered as the main impact on organizational effectiveness (Anderson et al., 2014). This study discovers important practical implications. Our results suggest that CQ could be beneficial to employees’ innovative behavior, both directly and indirectly. Therefore, organizations should adopt appropriate methods to manage employees effectively. First, this study encourages managers to test candidates’ CQ during the recruitment process and to preferentially select candidates with higher CQ. For example, in the process of recruitment, organizational managers can choose the candidates who get high scores in CQ measurement. Additionally, the organizations could also choose candidates who have good experiences communicating with individuals from different countries (Hu et al., 2019). This study also suggests that HR managers or leaders should act to increase employees’ cross-cultural competence such as CQ. In addition, it urges organizations develop HR practices in order to improve employees’ CQ. For example, Blasco et al. (2012) suggest enhancing cognitive CQ by in-depth study of the host country, whereas Engle and Crowne (2014) also suggest that discussing questions or concerns with a local “culture coach” can successfully increase team members’ CQ. A recent longitudinal pilot study also confirmed that CQ training can improve individual innovative work behavior (Azevedo and Shane, 2019). These programs could enable employees to understand different cultures, which in turn deal with types of situations in a diverse cultural setting (Chen et al., 2012). Finally, the findings also provide suggestions for organizations to establish a favorable organizational environment. Considering the high level of climate for inclusion in facilitating CQ-innovative behavior relationship, the organizations should make best efforts to foster inclusive climate. As previous study suggested (Davies et al., 2019), the organizations should consider the impact of national cultural values on building the inclusive climate, in particular, Korea, a country in which power distance, collectivism, and cultural tightness are relatively distinctive. Thus, the organizations may take advantage of organizational inclusion practices to improve employees’ perception of inclusion. For example, provide mechanisms for voice and communication and sharing within the workgroup and encourage participation in decision making and group discussion (Tang et al., 2015; Shore et al., 2018). Given the crucial impact of managers’ response on employee experiences of inclusion (Buengeler et al., 2018), it is essential that managers need to perform authentically and strategically implement inclusive-HR practices, with the goal of being inclusionary. Furthermore, managers could also foster an inclusive climate by supporting migrant workers as group members and encourages diverse contributions.

### Limitations and Future Research

Notwithstanding its contribution, our study has few limitations. First, following the method recommended by Podsakoff et al. (2012), we designed a two-wave study to control common method bias issue. However, the cross-sectional design of our study may not allow inferring causality. Thus, we recommend future research should conduct a longitudinal or multilevel design to examine the possible relationships between each variable. Second, this study measures CQ at overall level. However, the subdimensions of CQ aspects (e.g., metacognitive) are also especially important for understanding the cognitive complexity of cross-cultural individuals (Korzilius et al., 2017). Future study could consider the need for investigating of the relationship between CQ and innovative behavior from different subdimensions. Third, the sample size was not large. What is more, the generalizability of our findings may be a concern because of the use of Chinese migrant worker samples in Korea. As suggested by previous studies, team diversity and cultural distance may also affect the employee’s knowledge sharing (Chua et al., 2012; Bodla et al., 2018). However, this study was designed in a less cultural diversity case that may limit the generalizability of global cases. Therefore, future research should consider the potential impact of cultural differences and examine our hypothesized model in multicultural team settings using a larger sample. Fourth, the moderating variable may be considered as another limitation in our research. Previous researchers have argued for the appropriate level of organizational climate (Schneider et al., 2013) and indicated that it refers to the aggregate of the employee’s perception. Therefore, future studies may assess climate for inclusion with a multilevel research design to explore fruitful results. Finally, although this study considers climate for inclusion as a critical factor that may affect the relationship between CQ and innovative behavior, evidence shows that organizational factors such as organizational size, structure, organizational knowledge sharing (Kim and Park, 2015; Dy Bunpin et al., 2016), innovation climate (Ren and Zhang, 2015), and HR practices (Prieto and Pérez-Santana, 2014) also have a significant impact on individual innovative behavior. In addition, previous studies have indicated that interaction of cultural distance with CQ can affect individual’s behavior (Ng et al., 2019). Therefore, future research could examine other potential variables that might have an impact on innovative behavior in our model.

## Conclusion

Using a cross-sectional design, the current study shows that CQ is positively related to migrant workers’ innovative behavior. In particular, the results reveal that CQ has an indirect effect on innovative behavior *via* knowledge sharing. Furthermore, the results present a moderated mediation model, for which climate for inclusion moderates the mediating role of knowledge sharing in the relationship between CQ and migrant workers’ innovative behavior. This study can be beneficial to help us understand the mechanism of how cross-cultural competency (i.e., CQ) could facilitate innovative behavior. And the findings confirm the importance of CQ and its boundary context to innovative behavior, which provide a very promising framework for studying CQ and innovative behavior across cultures.

## Data Availability Statement

The raw data supporting the conclusions of this article will be made available by the authors, without undue reservation.

## Ethics Statement

Ethical review and approval was not required for the study on human participants in accordance with the local legislation and institutional requirements. Written informed consent from the participants was not required to participate in this study in accordance with the national legislation and the institutional requirements.

## Author Contributions

All authors listed have made a substantial, direct, and intellectual contribution to the work, and approved it for publication.

### Conflict of Interest

The authors declare that the research was conducted in the absence of any commercial or financial relationships that could be construed as a potential conflict of interest.
